# The evolution of HIV-1 reverse transcriptase in route to acquisition of Q151M multi-drug resistance is complex and involves mutations in multiple domains

**DOI:** 10.1186/1742-4690-8-31

**Published:** 2011-05-11

**Authors:** Jean L Mbisa, Ravi K Gupta, Desire Kabamba, Veronica Mulenga, Moxmalama Kalumbi, Chifumbe Chintu, Chris M Parry, Diana M Gibb, Sarah A Walker, Patricia A Cane, Deenan Pillay

**Affiliations:** 1 Virus Reference Department, Microbiology Services, Colindale, Health Protection Agency, London, UK; 2 UCL/MRC Centre for Medical Molecular Virology, Division of Infection and Immunity, UCL, Windeyer Institute, London, UK; 3 University Teaching Hospital, UNZA School of Medicine, Lusaka, Zambia; 4 MRC Clinical Trials Unit, London, UK

## Abstract

**Background:**

The Q151M multi-drug resistance (MDR) pathway in HIV-1 reverse transcriptase (RT) confers reduced susceptibility to all nucleoside reverse transcriptase inhibitors (NRTIs) excluding tenofovir (TDF). This pathway emerges after long term failure of therapy, and is increasingly observed in the resource poor world, where antiretroviral therapy is rarely accompanied by intensive virological monitoring. In this study we examined the genotypic, phenotypic and fitness correlates associated with the development of Q151M MDR in the absence of viral load monitoring.

**Results:**

Single-genome sequencing (SGS) of full-length RT was carried out on sequential samples from an HIV-infected individual enrolled in ART rollout. The emergence of Q151M MDR occurred in the order A62V, V75I, and finally Q151M on the same genome at 4, 17 and 37 months after initiation of therapy, respectively. This was accompanied by a parallel cumulative acquisition of mutations at 20 other codon positions; seven of which were located in the connection subdomain. We established that fourteen of these mutations are also observed in Q151M-containing sequences submitted to the Stanford University HIV database. Phenotypic drug susceptibility testing demonstrated that the Q151M-containing RT had reduced susceptibility to all NRTIs except for TDF. RT domain-swapping of patient and wild-type RTs showed that patient-derived connection subdomains were not associated with reduced NRTI susceptibility. However, the virus expressing patient-derived Q151M RT at 37 months demonstrated ~44% replicative capacity of that at 4 months. This was further reduced to ~22% when the Q151M-containing DNA pol domain was expressed with wild-type C-terminal domain, but was then fully compensated by coexpression of the coevolved connection subdomain.

**Conclusions:**

We demonstrate a complex interplay between drug susceptibility and replicative fitness in the acquisition Q151M MDR with serious implications for second-line regimen options. The acquisition of the Q151M pathway occurred sequentially over a long period of failing NRTI therapy, and was associated with mutations in multiple RT domains.

## Background

RT inhibitors (RTIs) are the mainstay of combination antiretroviral therapy (cART). Recommended first-line therapy regimens for HIV-1 treatment usually comprise two nucleos(t)ide RTIs (NRTIs) plus a third agent, either a non-nucleoside RTI (NNRTI) or a boosted protease inhibitor (bPI) or integrase inhibitor [1-3]. More than 90 mutations have been identified in HIV-1 RT to be associated with resistance to RTIs, and the majority are clustered either around the polymerase active site or the hydrophobic binding pocket of NNRTIs in the DNA pol domain of RT [4-7]. A consequence of some of these mutations is a severe loss of viral replicative capacity which can subsequently be restored by compensatory mutations elsewhere within RT [8].

The Q151M MDR is important because it has been shown to confer resistance to almost all NRTIs with the exception of TDF [9]. The Q151M MDR complex is composed of the Q151M mutation, which is normally the first to appear, followed by at least two of the following four mutations: A62V, V75I, F77L and F116Y [10]. The Q151M MDR complex was initially described to develop during long-term NRTI-containing combination therapy or NRTI therapy with zidovudine (AZT) and/or didanosine (ddI) [11,12]; however, it is now rarely observed in resource-rich countries, where more potent cART is used. It is believed that the Q151M MDR complex occurs infrequently because the Q151 to M mutation requires a 2-bp change (CAG to ATG), and the two possible intermediate changes of Q151L (CAG to CTG) and Q151K (CAG to AAG) significantly reduce viral replication capacity *in vitro *and are seldom observed *in vivo *[13-15]. The replicative capacity of a Q151L-containing virus was shown to improve in the presence of S68G and M230I mutations suggesting that compensatory mutations could favour the emergence of the Q151M MDR complex [13,15].

The Q151M complex has been identified in up to 19% of patients failing therapy containing stavudine (d4T) as part of ART rollout in the developing world, particularly where treatment is given without virological monitoring, thus allowing long term viraemia whilst on first-line therapy [16-18]. This includes the CHAP2 (Children with HIV Antibiotic Prophylaxis) prospective cohort study of Zambian children on a first-line therapy of lamivudine (3TC)/d4T/nevirapine (NVP) where 2 out of 26 children (8%) for whom resistance data were obtained had developed resistance via this pathway [19].

Although mutations causing resistance to RTIs have been shown to occur mainly in the DNA pol domain of RT, recent studies have implicated mutations in the C-terminal region of RT in resistance and possibly in restoring replication fitness of the HIV-1 drug-resistant variants [20,21]. Some of these mutations, such as N348I in the connection subdomain, have been reported to have a prevalence of 10-20% in treatment-experienced individuals [22]. The N348I mutation is associated with M184V and TAMs, and increases resistance to NRTIs such as AZT, as well as the NNRTI NVP. N348I confers resistance by reducing RNase H activity which allows more time for the excision or dissociation of the RT inhibitors [22-27]. However, few data are available on the evolution and genetic linkage of C-terminal mutations in the context of Q151M MDR complex, especially in non-B subtypes. In this study, we performed a detailed analysis of sequential samples collected from a patient in the CHAP2 cohort study who had developed resistance via the Q151M pathway to dissect the intrapatient viral population dynamics in the context of full-length RT.

## Results

We investigated the emergence of the Q151M MDR complex in one of the two patients in the CHAP2 cohort study who had developed resistance via the Q151M pathway [19]. The patient, designated P66, was infected with HIV-1 subtype C virus.

### Dynamics of emergence and genetic linkage of Q151M MDR complex mutations

**Figure 1:**
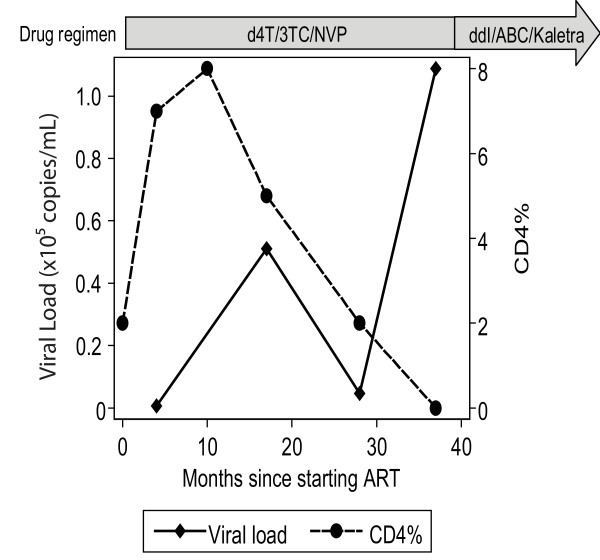
**Clinical profile of patient P66**. Longitudinal viral load, CD4% and ART regimen data for patient P66 during a 3-year follow up period starting from initiation of cART.

Patients enrolled in the CHAP2 cohort study had CD4 counts done approximately every 6 months and plasma was stored for retrospective viral load and genotypic testing. For patient P66, six samples were collected at 0, 4, 10, 17, 28, and 37 months after initiation of therapy; four of which were available for viral load testing and SGS analysis. The viral load and CD4% counts for patient P66 are shown in figure 1. We initially determined the development of Q151M MDR complex using SGS of full-length RT gene in the four sequential samples collected from patient P66 at 4, 17, 28 and 37 months. More than 30 single-genome sequences were generated per time point except for the 4- and 28-month time points when we obtained 6 and 0 sequences respectively. Genetic linkage analysis of the single genomes at 4, 17 and 37 months showed that the patient acquired the Q151M MDR mutations in the order: A62V, V75I and finally Q151M (Table 1). The emergence of Q151M after the secondary mutations A62V and V75I is rare. In addition, the analysis showed that drug resistance mutation T69N was genetically linked to Q151M MDR mutations and was acquired prior to Q151M.

**Table 1:** The sequential acquisition of Q151M MDR mutations and the frequency of other RT mutations linked to MDR mutations, in patient P66.

Type or Location of mutations	**Wild-type residue**^**a**^	Genetic linkage of other mutations to Q151M MDR			
		
		4 months (636)^b^		17 months (51,000)	37 months (108,769)
		
		n = 5^c^	n = 1	n = 33	n = 31
	A62	V		V	V
Q151M MDR	V75			I	I
	Q151				M

	T69			N^45^	N^100^
Other NRTI	M184	I^80^V^20d^	I^100^	V^100^	V^100^
	L210			S^6^F^3^	F^87^

	V90	I^20^		I^3^	
	E138	A^100^	A^100^	A^100^	A^100^
NNRTI	Y181	I^100^	I^100^	I^100^	I^100^
	H221			Y^70^	Y^100^
	M230		L^100^		
	N348	I^100^	I^100^	I^3^	

	I31			L^94^	L^100^
	A33				V^97^
	T48				S^100^
	S68				G^100^
	K102				R^61^
	S123				N^100^
Other DNA pol domain	I135	V^80^		L^58^V^18^T^15^	T^100^
	R174			K^18^	K^97^
	K197				E^87^
	V202			I^91^	I^100^
	E203			D^3^	D^100^
	V314				I^26^

	M357			R^18^L^3^	
	A371				T^23^
	T386			I^9^	I^100^
Other connection subdomain	E399			D^58^	D^100^
	A403	T^20^		T^45^	T^97^
	I458	V^20^	V^100^	V^24^	V^84^
	E471			D^39^	D^97^

RNase H domain	L517	I^60^	I^100^	I^56^	I^94^

^a ^Wild-type residue was determined based on 4-month sequences and frequency in treatment-naïve individuals as determined using Stanford University HIV database

^b ^Viral load in copies/mL

^c ^Number of single genomes linked or unlinked to Q151M MDR mutations

^d ^Percent of single genomes with that particular mutation calculated as follows: number of mutations per codon/number of single genomes linked or unlinked to Q151M MDR (n) × 100%

Accessory mutations in the DNA pol domain of RT have previously been demonstrated in the route to acquisition of Q151M MDR complex in subtype B viruses [12,28]. We, therefore, determined whether accessory mutations developed in this subtype C HIV-1 virus and whether the C-terminal region of RT played a role in the emergence of the Q151M MDR complex. The emergence and presence of mutations in DNA pol domain, connection subdomain and RNase H domain were assessed by SGS, and their genetic linkage to Q151M MDR mutations was determined. A pre-treatment sample was not available for analysis from patient P66; therefore a codon change was scored as a mutation if it met one of the following criteria: (i) if it was a known drug resistance mutation as determined by International AIDS Society-USA (IAS-USA) [29], (ii) if it was not present in sequences from a previous time point or underwent a significant change in frequency between time points. This analysis showed a cumulative increase in mutations in all RT domains (Table 1). Mutations were identified at 12 codon positions in DNA pol domain, namely, 31, 33, 48, 68, 102, 123, 135, 174, 197, 202, 203 and 314; seven in connection subdomain, 357, 371, 386, 399, 403, 458 and 471; and one in RNase H domain, 517. The correlation between the progressive increments in the frequency of these mutations and the sequential acquisition of the Q151M MDR mutations suggested that they could be facilitating the emergence of the Q151M MDR complex. This notion is further supported by the observation that 18 out of the 20 mutations were present in a majority of the single genomes by 37 months and nearly half of them were present in all the single genomes (Table 1).

The Q151M MDR mutations were also genetically linked to NRTI mutations M184IV and L210F, and NNRTI mutations E138A, Y181I and H221Y (Table 1). Of note, the N348I mutation was identified in the connection subdomain of all single genomes at 4 months. However, the mutation was present in only one out of 33 single genomes at 17 months but none of the 31 single genomes at 37 months when the Q151M mutation emerged (Table 1).

### Intrapatient viral genetic diversity in the route to acquisition of Q151M MDR complex

**Figure 2:**
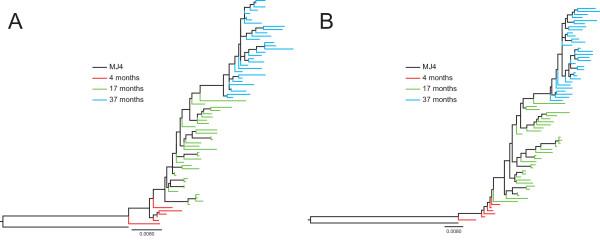
**ML phylogenetic analysis of single genome sequences**. Branch lengths were estimated using the GTR model of substitution and are drawn in scale with the bar at the bottom representing 0.008 nucleotide substitutions per site. The colour of each tip branch represents the time after initiation of therapy when the sample from which the single-genome originates was collected as shown in the legend in each figure. (A) Phylogenetic tree of 70 single genomes generated from 3 sequential samples from patient P66 infected with subtype C HIV-1 virus. (B) Same as (A) but with the following 12 RT drug resistance codons removed from the aligned single-genome sequences to determine the effect of drug resistance mutations on viral evolution: 62, 69, 75, 90, 138, 151, 181, 184, 210, 221, 230 and 348. The trees were rooted using the subtype C reference sequence MJ4.

The evolution and viral population dynamics within patient P66 were examined further by phylogenetic analyses. Maximum likelihood (ML) trees of the PR-RT single-genome sequences generated from the sequential samples of the patient are shown in Figure 2A. In general, the ML-inferred genealogy clustered all single genomes from each time point within a monophyletic clade with corresponding progressive increases in genetic distances. Intriguingly, the analyses also showed a serial replacement effect with sequences from successive time points arising from a single branch of a cluster of sequences from a preceding time point. This suggests a serial founder effect in the development of Q151M MDR. Furthermore, ML-inferred genealogy of the sequences with drug resistance codons removed showed that the serial founder effect and monophyletic clustering of the sequences from each time point was maintained (Figure 2B). This indicates that the identified accessory mutations could be playing an important role in the evolution and development of the Q151M MDR.

### High prevalence of some of the identified accessory mutations in subtype B and C infected patients

Next, we determined if the 20 accessory mutations that we identified in patient P66 were present in other patients who had developed resistance via the Q151M pathway. We compared mutation frequencies in subtype B or C samples from RTI-treatment naïve patients and Q151M-containing patient samples on the Stanford University HIV drug resistance database. A significant number of sequences (15 to 12,361) were available for analysis in each subgroup, except for connection subdomain and RNase H domain of Q151M-containing subtype C sequences, in which there was only one sample sequenced beyond the DNA pol domain. Therefore, the analysis for subtype C sequences could only be carried out for the DNA pol domain. This showed that eight out of the 12 codon positions identified in the DNA pol domain of patient P66 were significantly associated with the sequences containing the Q151M mutation compared to RTI-treatment naïve sequences. These codon positions were 31, 33, 48, 68, 123, 174, 202 and 203 (*P *≤ 0.042; Table 2). In contrast, two of these codon positions, namely 48 and 174, were not associated with the acquisition of Q151M in subtype B infected patients, but an additional two others were, namely 102 and 197 (*P *≤ 0.029). Interestingly, codon positions 386 and 403 in connection subdomain were also significantly associated with the acquisition of Q151M in subtype B infected individuals (*P *≤ 0.018). These data indicate that some of the accessory mutations identified in the DNA pol domain and connection subdomain of patient P66 are highly prevalent in patients who develop resistance through the Q151M pathway and that they could be playing an important role in the acquisition of the Q151M MDR.

**Table 2:** Analysis of the frequency of accessory mutations in RTI-treatment naïve and Q151M-containing sequences on Stanford University HIV database.

	Subtype C						Subtype B					
	
		RTI-treatment naïve		**Q151M**^**b**^				RTI-treatment naïve		Q151M		
						
RT domain	Wild-type C^a^	No. of seqs.^c^	% mut. freq.^d^	No. of seqs.	% mut. freq.	Mut.% Diff.^e^	Wild-type B	No. of seqs.	% mut. freq.	No. of seqs.	% mut. freq.	Mut.% Diff.
	**I31**	3,557	<1	24	4 (L)	+4	**I31**	10,329	<1	373	5 (RL)	+5
	**A33**	3,600	<0.1	24	4 (V)	+4	**A33**	10,388	<1	375	2 (V)	+2
	**T48**	3,941	15 (SE)	44	39 (S)	+24	S48	12,361	3 (T)	492	2 (T)	-1
	**S68**	3,998	<1	44	73 (G)	+73	**S68**	12,350	4 (G)	491	50 (GNRK)	+46
	K102	4,004	2 (Q)	44	5 (QN)	+3	**K102**	12,204	5 (QR)	492	8 (QR)	+3
	**D123**	3,757	62 (SGNE)	44	77 (SGN)	+15	D123	12,001	29 (ENS)	492	28 (EN)	-1
DNA pol	I135	3,942	28 (TVR)	44	23 (TVMK)	-5	**I135**	11,994	43 (TVLR)	492	38 (TVLMR)	-5
	**Q174**	3,851	39 (KR)	44	61 (KR)	+22	Q174	12,241	7 (KEHR)	492	9 (RKH)	+2
	Q197	3,999	3 (K)	44	2 (E)	-1	**Q197**	12,316	3 (KE)	492	5 (EK)	+2
	**I202**	3,955	7 (V)	44	27 (V)	+20	**I202**	12,151	9 (V)	492	24 (V)	+15
	**E203**	4,008	1	44	7 (K)	+6	**E203**	12,304	1	492	10 (DK)	+9
	V314	1,889	2 (A)	19	0	-2	V314	4,332	<1	91	0	0

	M357	715	33 (RKLVIT)	1	100 (K)	NC^f^	M357	1,481	31 (TKVIR)	75	33 (TVRKI)	+2
	A371	684	6 (V)	1	0	NC	A371	1,518	5 (V)	75	11 (VT)	+6
	T386	657	11 (IV)	1	100 (I)	NC	**T386**	1,504	18 (IV)	75	49 (IAVSPM)	+31
connection	E399	595	5 (DG)	1	0	NC	E399	1,381	14 (D)	75	13 (DG)	-1
	T403	556	6 (MASI)	0	NA^g^	NA	**T403**	744	23 (MISAVL)	17	0	-23
	V458	401	6 (I)	0	NA	NA	V458	651	1 (I)	16	0	-1
	E471	396	3 (D)	0	NA	NA	D471	658	3 (EN)	16	0	-3

RNase H	L517	392	7 (I)	0	NA	NA	L517	636	15 (IV)	15	0	-15

^a^The residue occurring in the majority of RTI-treatment naïve patient sequences is referred to as wild-type. Codon positions showing statistically significant difference in mutation frequency between RTI-treatment naïve and Q151M-containing sequences are indicated in bold. Subtype C: I31 (*P *= 0.033), A33 (*P *= 0.024), T48 (*P *< 0.0001), S68 (*P *< 0.0001), D123 (*P *= 0.042), Q174 (*P *= 0.003), I202 (*P *< 0.0001) and E203 (*P *= 0.011). Subtype B: I31 (*P *< 0.0001), A33 (*P *= 0.024), S68 (*P *< 0.0001), K102 (*P *= 0.006), I135 (*P *= 0.029), Q197 (*P *= 0.015), I202 (*P *< 0.0001), E203 (*P *< 0.0001), T386 (*P *< 0.0001) and T403 (*P *= 0.018).

^b^Sequences containing the Q151M mutation

^c^The number of sequences used for the analysis. Only one sequence was used per individual if multiple sequences were available.

^d^The percentage of sequences with an amino acid change from wild-type residue. The mutant amino acid(s) present at a frequency greater than 1% is shown in brackets.

^e^The difference in mutation frequency between Q151M-containing and RTI-treatment naïve sequences; plus sign indicates an increase and minus sign a decrease in mutation frequency in Q151M-containing sequences compared to RTI-treatment naïve.

^f^NC = Not calculated (one sequence available for analysis).

^g^NA = Not applicable (no sequences available for analysis).

### C-terminal mutations are not associated with decreased susceptibility of Q151M-containing viruses to NRTIs in patient P66

**Figure 3:**
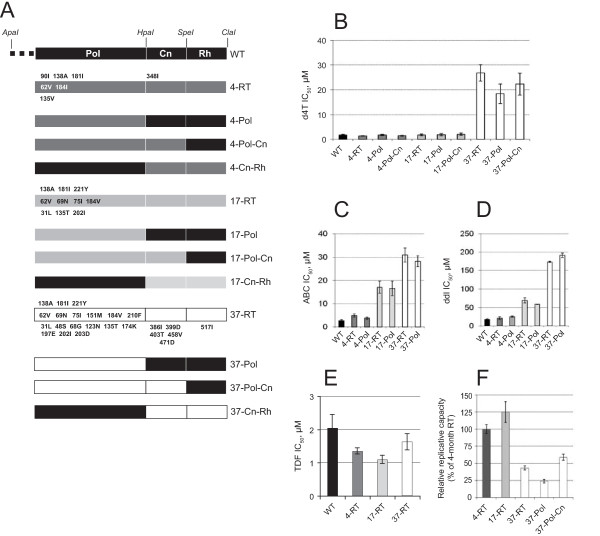
**NRTI susceptibilities and replicative capacity associated with RT domains of patient P66**. (A) Schematic representation of full-length and chimeras of subtype C wild-type and patient-derived RT *gag-pol *expressing vectors used for drug susceptibility and replicative capacity testing. The positions of the restriction sites used for cloning of patient-derived PR-RT fragments (ApaI and ClaI) and for RT domain swapping (HpaI and SpeI) are indicated above the vector. The origins of the RT domains are shown as different coloured boxes: black, wild-type virus; dark gray, patient-derived RT at 4 months; light gray, patient-derived RT at 17 months; and white, patient-derived RT at 37 months. The names of the vectors are indicated on the right with a number representing the month when the sample was collected followed by the patient-derived domain(s) being expressed. Mutations present in each domain are shown on the full-length RT constructs as follows: inside the box, NRTI-associated resistance mutations; above the box, NNRTI-associated resistance mutations; and below the box, other mutations. Pol, DNA pol domain; Cn, Connection subdomain; Rh, RNase H domain. (B) Susceptibility to d4T exhibited by patient-derived full-length RTs and RT domains. (C) Susceptibility to second-line NRTI ABC exhibited by patient-derived full-length RTs. (D) Susceptibility to second-line NRTI ddI exhibited by patient-derived full-length RTs. (E) Susceptibility to TDF exhibited by patient-derived full-length RTs. (F) Replicative capacities relative to virus expressing full-length patient-derived RT from 4-months after initiation of therapy, set at 100%, are shown for each virus. The error bars represent standard error of the mean of three or more independent experiments.

Consequently, we investigated whether the C-terminal mutations we observed affected susceptibility to NRTIs. Unique restriction sites were introduced in RT and IN genes without changing the amino acid coding, in both the packaging vector and cloned patient fragments in order to facilitate RT domain-swapping (Figure 3A). The patient-derived RTs remained d4T-susceptible until the development of the Q151M mutation at 37 months, when there was a significant increase (~16-fold) in IC_50 _values compared to wild-type RT (Figure 3B; *P *< 0.002). At most we observed a 1.3-fold change in susceptibility to d4T at 4 or 17 months leading us to conclude that Q151M is the main contributor to d4T resistance in the Q151M MDR complex. The patient-derived RT exhibited a 23-fold increase in 3TC IC_50 _values at 4 months which did not increase at 17 and 37 months despite the acquisition of the Q151M MDR mutations (Table 3). The effect on susceptibility to 3TC was probably due to M184I/V mutations which were seen by 4 months. The 23-fold reduction in susceptibility is relatively lower than observed in other studies [30,31]. This could be because our assay uses full-length RT fragments derived from clinical isolates. It has recently been shown that the use of a co-evolved or subtype-specific C-terminal region of RT can influence the magnitude of drug resistance observed in a phenotypic drug susceptibility assay [32].

**Table 3:** 3TC, AZT and FTC susceptibilities associated with RT domains of patient P66.

Virus	3TC		AZT		FTC	
	
	**IC**_**50**_^**a**^	**FC**^**b**^	**IC**_**50**_^**a**^	**FC**^**b**^	**IC**_**50**_^**a**^	**FC**^**b**^
Wild-type	8.5 ± 0.8		168.6 ± 46.8		2.2 ± 0.3	
4-RT	198.8 ± 18.6	23.3	76.9 ± 6.8	0.5	184.2 ± 14.2	84.9
4-Pol	211.5 ± 17.5	24.8	60.0 ± 13.8	0.4	168.1 ± 6.4	77.5
17-RT	224.1 ± 16.9	26.3	56.4 ± 7.2	0.3	228.8 ± 6.7	105.5
17-Pol	206.5 ± 9.7	24.2	58.1 ± 14.2	0.3	218.9 ± 13.3	100.9
37-RT	219.7 ± 7.5	25.8	5120.9 ± 515.6	30.4	230.9 ± 10.2	106.4
37-Pol	217.8 ± 18.1	25.6	2025.3 ± 144.2	12.0	231.5 ± 17.1	106.7

^a^50% inhibitory concentration in nM ± SEM.

^b^Fold change in IC_50 _compared to wild-type virus.

Analysis of susceptibilities of patient-derived RTs to the CHAP2 second-line NRTIs ddI and ABC showed a cumulative decrease in susceptibility in the order; 1.2- and 1.7-fold at 4 months, 4- and 6-fold at 17 months, and finally 9.9- and 10.8-fold at 37 months, respectively (Figure 3C). Thus, unlike d4T the cumulative acquisition of mutations on the route to Q151M MDR complex results in a parallel cumulative decrease in susceptibilities to ABC and ddI. In addition, the recombinant viruses expressing patient-derived RTs exhibited decreased susceptibilities to NRTIs FTC of >79-fold at 4 months and AZT of >15-fold at 37 months (Table 3) but remained susceptible to TDF even after the acquisition of the Q151M mutation at 37 months (Figure 3D) with no significant increases in IC_50 _values (*P *> 0.18). The susceptibility to TDF could probably be influenced by the presence of M184V which has been shown to increase HIV-1 sensitivity to TDF [33,34].

The expression of the patient-derived DNA pol domain at 37 months plus wild-type C-terminal region or coevolved connection subdomain showed no significant differences in IC_50 _values to d4T (*P *> 0.05) suggesting that none of the identified C-terminal mutations in patient P66 at 37 months contributed to the reduction in susceptibility to d4T (Figure 3B). Similarly, the coevolved C-terminal region did not contribute to 3TC resistance, including the previously identified N348I mutation at 4 months, neither did they contribute to the decreases in susceptibility to ABC, ddI or FTC (Figure 3C and 3D and Table 3). However, we observed an effect of the C-terminal mutations at 37 months to AZT, with the co-evolved C-terminal region contributing a 2.5-fold increase in AZT resistance (Table 3).

Finally, we determined the effect of the mutations on susceptibility to NVP, the NNRTI used for first-line therapy in the CHAP2 cohort study. The recombinant viruses expressing the patient-derived C-terminal region at 4 months, but not at 17 or 37 months, exhibited a 5-fold increase in the NVP IC_50 _value relative to wild-type (*P *< 0.002; Table 4). The decrease in NVP susceptibility associated with the C-terminal domain at 4 months is likely due to the presence of the N348I mutation in the connection subdomain which disappears at later time points.

**Table 4:** NVP susceptibilities associated with RT domains of patient P66.

Virus	**IC**_**50**_^**a**^	**FC**^**b**^
Wild-type	86.47 ± 11.84	

4-RT	>6,000	>66
4-Pol	>6,000	>66
4-Pol-Cn	>6,000	>66
4-Cn-Rh	410.5 ± 55.2	4.7

17-RT	>6,000	>66
17-Pol	>6,000	>66
17-Pol-Cn	>6,000	>66
17-Cn-Rh	73.83 ± 8.54	0.9

37-RT	>6,000	>66
37-Pol	>6,000	>66
37-Pol-Cn	>6,000	>66
37-Cn-Rh	88.23 ± 12.95	1.0

^a^50% inhibitory concentration in nM ± SEM.

^b ^Fold change in IC_50 _compared to wild-type virus.

### Connection subdomain mutations in patient P66 partially restore replicative fitness of Q151M MDR-containing viruses

Since we did not observe any association of C-terminal mutations at 37 months with a decrease in susceptibilities to first-line drugs, we evaluated their effect on virus replicative capacity by infecting HEK293T cells with equivalent amounts of virus. The patient's sample before initiation of therapy was not available, thus the replicative capacity of the viruses measured by relative luciferase light units was compared to that of the virus expressing full-length patient-derived RT at 4 months. The patient-derived RT at 4 months had already developed the M184I mutation which is known to affect viral replicative fitness [35,36]. The virus expressing the full-length patient-derived RT containing the Q151M mutation at 37 months demonstrated ~42% replicative capacity of full-length patient-derived RT at 4 months (*P *< 0.0001; Figure 2E). This was further significantly decreased to ~22% (*P *< 0.0001) when the patient-derived DNA pol domain at 37 months was expressed in combination with wild-type connection subdomain and RNase H domain. This decrease in replicative capacity was fully compensated (to ~55% replicative capacity) by the coexpression of the coevolved connection subdomain at 37 months. In contrast, replicative capacity of the full-length patient-derived RT at 17 months was comparable to that at 4 months. This suggests that the Q151M mutation, as well as being the main determinant of drug resistance in the Q151M MDR complex, also has a more significant effect on virus replication fitness that is partially restored by mutations in the connection subdomain.

## Discussion

Multiple mutations throughout HIV-1 RT are associated with RTI resistance including recently identified mutations in the connection subdomain and RNase H domain [10,21,27]. However, there are few data on sequential acquisition and genetic linkage of these mutations and their impact on drug susceptibility and replicative capacity, especially in non-B subtype HIV-1 viruses which account for nearly 90% of the epidemic worldwide [37]. In this study, we took advantage of treatment failure in the absence of viral load-guided therapy to dissect the relative contribution of RT domains in the route to high-level NRTI drug resistance through the Q151M pathway.

As expected we found that the development of mutations was broad throughout RT. The virus from the patient we investigated had developed more than 12 known drug resistance mutations and 20 additional mutations in RT, nearly half of which were located in the connection subdomain. A refined analysis of the emergence and development of these mutations in sequential samples by SGS revealed a chronological increase in frequency that paralleled the sequential acquisition of Q151M MDR mutations. In addition, the analysis showed genetic linkage of most of these mutations to Q151M MDR mutations indicating an association between the two. Although our results are from one patient, the identified mutations in the pol domain at codon positions 68 and 202 were previously identified in patients infected with subtype B HIV-1 viruses [12,28] and in an HIV database sequence analysis done in this study (Table 2). The database sequence analysis also showed that the DNA pol domain mutations at codon positions 31, 33, 48, 102, 123, 135, 174, 197 and 203 were significantly associated with Q151M in subtype B and/or C.

We show that although the connection subdomain mutations were acquired in parallel with Q151M MDR mutations they were not directly associated with drug resistance but played a role in improving the replicative fitness of the Q151M-containing viruses. Our findings confirm previous reports showing that the Q151M-containing virus replicates poorly [13,14,38,39]. We clearly show that the patient-derived connection subdomain is important for improving the Q151M-containing virus' replicative fitness and is thus important for the development of the Q151M pathway. It will be interesting to elucidate the particular mutations involved and the mechanism behind the connection subdomain's effect on replicative fitness of the Q151M-containing RT. The mutation at connection subdomain codon positions 386 and 403 were significantly associated with Q151M in the subtype B database analysis; however, a similar analysis could not be carried out for subtype C due to lack of samples sequenced beyond the DNA pol domain. Since the connection subdomain is involved in positioning of the template-primer complex at the polymerase active site, one possibility could be that the mutations improve enzyme-substrate interactions at the active site. Of note, the intermediate Q151K or L mutations which have been postulated to be involved in the emergence of the Q151M mutation were never identified in our SGS analysis. It is possible that these mutations do emerge but are only present transiently due to their negative effect on replication and, as a result, were missed in this analysis. This possibility could not be explored further in this study as we were unable to amplify any genomes at 28 months, the time point prior to the emergence of the Q151M mutation.

It was surprising to observe that the patient-derived connection subdomain and RNase H domain were not associated with the decreased susceptibility to NRTIs exhibited by the Q151M MDR-containing RTs and also that the N348I mutation disappeared prior to the acquisition of Q151M. As described earlier, N348I confers drug resistance by decreasing RNase H activity, thus it will be interesting to explore if a negative correlation exists between reduced RNase H activity and Q151M. Another surprising finding was that full-blown resistance did not develop until 37 months after initiation of therapy, even though the viral load had been relatively high at earlier time points. This raises the possibility of suboptimal use of the drugs contributing to the emergence of the Q151M MDR complex.

## Conclusions

Understanding the evolution and molecular mechanisms leading to the emergence of the Q151M MDR complex is important especially in light of its relatively frequent occurrence in some ARV rollout cohorts. As shown in this study and other previous reports [9], the presence of the Q151M mutation significantly limits the options for second-line therapies as the Q151M-containing virus remains only susceptible to one approved NRTI, TDF. Our results showed that the Q151M MDR takes a long time to develop and keeping patients on failing NRTI therapy could be facilitating its emergence. The Q151M MDR is also often linked to other NRTI and NNRTI mutations which develop earlier and thus further limiting the options for second-line regimens. In addition, the virus acquires compensatory mutations throughout RT which make it fitter, resulting in a virus that could persist even after switching to second-line therapy. This is a major obstacle in the developing world where fixed second-line therapies are composed of two alternate NRTIs (usually not TDF) and bPI. Thus, these types of studies are important in guiding public health approaches to the treatment and clinical management of HIV-1 infections in resource-poor settings.

## Methods

### Clinical HIV samples and database analysis

The plasma samples characterized in this study were from a patient enrolled in the CHAP2 prospective cohort study at the University Teaching Hospital in Lusaka, Zambia [19]. Children in this study were initiated on first-line cART of 3TC/d4T/NVP (adult Triomune30) and, following immunological or clinical failure, were switched to a fixed second-line therapy of Abacavir (ABC)/ddI/Kaletra. The prevalence of identified accessory mutations in clinical samples was analyzed using the Stanford University HIV drug resistance database (http://hivdb.Stanford.edu).

### SGS assay

A previously described SGS assay [40] was modified by designing new antisense primers in integrase (IN) and used to sequence the full-length protease (PR) and RT genes from sequential samples. Briefly, viral RNA was extracted from 200 μL of plasma using QIAmp UltraSens Virus Kit (Qiagen) following manufacturer's instructions and eluted in 60 μL of elution buffer. cDNA synthesis and single genome PCR reactions were carried out as described previously [40] using primers 1849+ (5'-GATGACAGCATGTCAGGGAG-3') and 4368- (5'-GCTAGCTACTATTTCTTTTGCTACT-3'), followed by a nested PCR with primers 1870+ (5'-GAGTTTTGGCTGAGGCAATGAG-3') and 4295- (5'-CTTTCATGCTCTTCTTGAGCCT-3'). Positive PCR products were identified by agarose gel electrophoresis and purified using illustra GFX PCR DNA and Gel Band Purification Kit (GE Healthcare), and sequenced by the dideoxy ABI sequencing systems in both directions using overlapping internal primers. Sequences were analyzed using Sequencher software (Gene Codes) and aligned by using subtype-specific consensus sequences. Any sequences containing double peaks in the chromatographs were excluded. Drug resistance mutations were defined by using the Stanford University HIV drug resistance database.

### Phylogenetic analyses

Full-length PR-RT nucleotide single-genome sequences from patient P66 and subtype-specific reference sequence MJ4 (subtype C) were aligned using Clustal W in MEGA4 software[41]. The aligned sequences were imported into PhyML tree building software and ML trees were constructed using the GTR model and the robustness of the trees was evaluated by bootstrap analysis with 500 rounds of replication.

### Single-replication cycle drug susceptibility assay

A recently described three plasmid-based retroviral vector system using a luciferase reporter gene was used to study phenotypic drug susceptibility [42,43]. Briefly, vector p8MJ4 was modified to accommodate RT domain-swapping by introducing three restriction enzyme sites, HpaI (flanking RT amino acids 288/289), SpeI (flanking RT amino acids 423/424) and ClaI (flanking IN amino acids 4/5) creating p8MJ4-HSC. The MJ4 sequence also contains a natural and unique ApaI site in p6 region of gag. In addition, the SpeI site in gag and two ClaI sites (upstream of gag initiation codon and in gag) were eliminated to ensure that the introduced sites were unique. In parallel, patient-derived PR-RT single genomes that closely represented the sequence of the majority of the single genomes at each time point were subcloned into a TOPO-TA vector (Invitrogen) by PCR using primers GagApaF (5'-GCAGGGCCCCTAGGAAAAAGGGC-3') and CRhINClaIR1 (5'-CCTTATCGATTCCATCTAGAAATAGC-3'). Similarly, HpaI (flanking RT amino acids 288/289) and SpeI (flanking RT amino acids 423/424) sites were introduced and any HpaI or SpeI sites that were present in the cloned patient fragments were removed using sequence-specific primers. Mutagenesis reactions were carried out by site-directed mutagenesis using QuikChange Lightning Multi Site-Directed Mutagenesis Kit (Agilent Technologies) and the presence and absence of each mutation was verified by sequencing. The other two vectors used in the system are pMDG encoding the vesicular stomatitis virus G protein and retroviral expression vector pCSFLW which encodes for the luciferase reporter gene. Virus stocks were prepared by cotransfection of HEK293T cells as described previously [44-46], diluted 50- to 500-fold and used to infect HEK293T target cells. The virus and target cells were incubated with medium containing varying drug concentrations for 48 h. Infectivity was determined by measuring luciferase activity in the target cells using Steady-Glo reporter assay system (Promega). Data were expressed relative to that of no drug controls and the drug concentrations required to inhibit virus replication by 50% (IC_50_) were determined by linear regression analysis. Results are expressed as fold changes in the IC_50 _compared to wild-type subtype C virus.

### Antiretroviral drugs

The NRTIs ABC, AZT, ddI, emtricitabine (FTC), 3TC and d4T; and the NNRTIs efavirenz (EFV), etravirine (ETV), and NVP were obtained from the NIH AIDS Research and Reference Reagent Program. TDF was a generous gift from Gilead Sciences (Foster City, CA, USA).

### Replicative capacity Assay

Recombinant viruses expressing wild-type and patient-derived RT domains were normalized for p24 capsid (Genetic Systems HIV-1 Ag EIA; Bio-Rad) and used to infect target HEK293T cells in a single-cycle-replication assay. Replicative capacity was determined by measuring luciferase activity as described above.

### Statistical analyses

Student's *t *test was used to describe differences in IC_50 _values and replicative capacity and two proportions analysis was performed by using Fisher's Exact test with *P *values < 0.05 regarded as significant for both tests (StataSE software).

### Nucleotide sequence accession numbers

The single-genome sequences generated and used in this study have been submitted to GenBank and assigned the accession numbers HQ111194-HQ111338.

## Competing interests

The authors declare that they have no competing interests.

## Authors' contributions

JLM carried out the bulk of the laboratory work, planning the study and writing the manuscript. RKG, CMP, DMG, ASW, PAC and DP were involved in planning the study, undertaking laboratory work and editing the manuscript. DK, VM, MK, CC, DMG were involved in undertaking clinical support work. All authors read and approved the final manuscript.
